# Correction: Human cytomegalovirus IE1 alters the higher-order chromatin structure by targeting the acidic patch of the nucleosome

**DOI:** 10.7554/eLife.15893

**Published:** 2016-03-14

**Authors:** Qianglin Fang, Ping Chen, Mingzhu Wang, Junnan Fang, Na Yang, Guohong Li, Rui-Ming Xu

Fang Q, Chen P, Wang M, Fang J, Yang N, Li G, Xu R-M. 2016. Human cytomegalovirus IE1 alters the higher-order chromatin structure by targeting the acidic patch of the nucleosome. *eLife* **5**:e11911. doi: 10.7554/eLife.11911.

Published 26 January 2016

In the second sentence of the abstract, the text ‘and discovered that IE1-CTD specifically interacts with the H2A-H2B acidic patch and impairs the compaction of higher-order chromatin structure’ has been replaced with ‘and discovered that the specific interaction between IE1-CTD and the H2A-H2B acidic patch impairs the compaction of higher-order chromatin structure’.

In the second paragraph of the Introduction, the text ‘is responsible for the association with the mitotic chromosome’ has been replaced with ‘is responsible for the association with the mitotic chromosome, specifically through the acidic patch of the nucleosome’.

The article has been corrected accordingly.

